# Endocrine‐Taste Crosstalk: A Scoping Review on Thyroid Dysfunction and Its Genetic Links to Taste Receptors With Dysgeusia

**DOI:** 10.1155/ije/1156681

**Published:** 2026-04-01

**Authors:** Panchami Pai, Ananya Chadaga, Srikant Natarajan, Chetana Chandrashekar, Gabriel Rodrigues, Sunitha Carnelio

**Affiliations:** ^1^ Department of Oral and Maxillofacial Pathology, Manipal College of Dental Sciences, Manipal Academy of Higher Education, Manipal, India, manipal.edu; ^2^ Department of Oral and Maxillofacial Pathology, Manipal College of Dental Sciences Mangalore, Manipal Academy of Higher Education, Manipal, India, manipal.edu; ^3^ Aster Al Raffah Hospital, Sohar, Oman

**Keywords:** altered tastes, taste receptor mutations, taste receptor polymorphism, thyroid dysfunction

## Abstract

Thyroid disorders are a common endocrine condition globally, affecting various bodily systems and metabolism. Changes in thyroid function affect smell and taste; yet, many patients remain unaware of their dysosmia and dysgeusia. Currently, there is a lack of detailed information regarding changes in physiological taste thresholds across different taste modalities or the genetic polymorphism(s) associated with them. This review underscores the association between genetic polymorphism in taste receptor genes with altered taste sensation in thyroid disease. Data bases from 1990 to 2025 were searched with appropriate Medical Subject Headings (MeSH) terms and Boolean operators (PubMed‐MEDLINE, Google Scholar, Scopus, Web of Science, and Embase). A total of 446 titles were retrieved. After elimination and thorough review, 18 articles were included, of which 3 articles met the inclusion and exclusion criteria. Understanding genetic changes in individuals with thyroid dysfunction can raise clinician awareness in identification and take proactive measures to educate, screen, and chart treatment protocols. Studies on agonist considered to be the novel drug targets can act on these taste receptors or those resistant to conventional drugs in the treatment of thyroid dysfunction opens new avenues for understanding thyroid regulation and potential drug development targeting Taste 2 Receptors (TAS2Rs).

## 1. Introduction

Taste perception is an important sensory function that mirrors overall health, helps in relishing food, and supports proper nutrition. Alteration in taste (dysgeusia) can lead to decreased appetite, malnutrition, diminished quality of life (QOL) and experience irritability, anxiety related to eating, reduced liking for food, and withdrawal from social interaction [1]. However, taste disorders have traditionally been linked to local, oral, or neurological disorders; recent clinical evidence suggests that systemic diseases, particularly endocrine disorders such as thyroid disorders, also impact physiological taste function. Thyroid hormones (T3, T4, and thyroid stimulating hormone [TSH]) regulate cellular metabolism, growth, neurodevelopment, and physiological homeostasis. They are known to affect growth and maintenance of taste bud cells through receptor interactions that modulate gene transcription, thereby influencing the turnover rate and sensitivity of taste receptor cells [2–4]. A link has been proven between bitter taste perception via TAS2R that play a vital role in modulating thyroid hormones and gland function [5, 6]. Hypo‐ and hyperthyroidism, the two commonly occurring thyroid disorders exert widespread systemic effects, including alterations in taste sensory perception such as changes in overall taste sensitivity and impaired ability to distinguish between different taste modalities [7]. Multiple factors, including changes in the salivary environment, damage to peripheral nerve endings, and changes in receptor activity with molecular and genetic changes in taste receptor expression within taste buds, are most significant. The decreased level of T3 and T4 hormones potentially impair the turnover and maintenance of the taste cells that could delay or reduce the response to taste and can influence the actions of sensory neurons, whereby a deficit could also compound the alteration at the neural level of the gustatory pathways [2, 3].

Symptoms of dysgeusia in patients with thyroid diseases vary and a metallic taste along with xerostomia could be a warning sign. Saliva plays a vital role in taste perception by dissolving tastants and helping them interact with taste receptors. Taste transduction heavily depends on its composition and flow rate, so variations in these can impact the process [7, 8].

Interestingly, genetic changes linked to taste dysfunction in thyroid disorders, such as TAS2R38 polymorphisms, differentiate the taste between phenylthiocarbamide (PTC) and 6‐n‐propylthiouracil (PROP) [9]. As a result, altered taste perception could serve as an early and sensitive indicator of mild or subclinical thyroid dysfunction, especially in individuals genetically predisposed to alterations in taste or taste sensitivity.

The current review aims to shed light on the association between genetic variants, especially single‐nucleotide polymorphisms (SNPs) in taste receptor genes, with dysgeusia and thyroid diseases, and the potential efficacy of various agonists/drugs that modulate thyroid hormone activity.

## 2. Methods

### 2.1. Study Design

This review followed guidelines based on the PRISMA extension for scoping reviews (PRISMA‐ScR) 2018 checklist [10] and Askey and O’Malley framework, incorporating methodological guidance for scoping reviews by the authors in [11] and was done by defining the research question, identifying suitable studies, specifying the criteria for inclusion and exclusion, extracting relevant information, compiling the findings and tabulating the results.

### 2.2. Search Strategy

The literature review was conducted using electronic databases (PubMed, Web of Science, Scopus, Embase, and Google Scholar) employing combinations of the keywords such as taste receptor polymorphism, altered tastes, mutations in taste receptors, genotypes, thyroid dysfunction, and thyroid disease, combined with appropriate Boolean operators (Supporting file: S1 and Table 1). Additionally, a manual search was performed to find relevant literature from 1990 to 2025. Articles other than English were translated and reviewed [12]. Duplicates were removed before further analysis.

**TABLE 1:** Inclusion and exclusion criteria to minimize heterogeneity.

Inclusion criteria	Exclusion criteria
• Cross‐sectional, case‐control, or cohort design reporting the association between taste receptor polymorphism, altered taste in patients with thyroid dysfunctions	• Articles associated with altered taste or taste receptors not associated with thyroid dysfunction or other causes such as animal studies
• Patients recruited clinically, relevant lab reports and pathologically confirmed thyroid dysfunction cases	• Studies reporting overlapping data, case reports, case series
• In vivo as well as in vitro studies	• Unusable or insufficient genotype data reported
	• Abstracts, review articles, letters to the editor, short communications, conference papers

### 2.3. Data Extraction With Evidence

Two independent investigators assessed data extraction from the eligible studies following the inclusion/exclusion criteria listed in Table 1. All records were imported into the Rayyan software [13]. Deduplication was performed. Disagreement between the two reviewers on any item was solved with the assistance of a third adjudicator, leading to unanimous consent. Two reviewers performed full‐text (FT) screening and data extraction and independently screened each article for the FT. The extracted data were entered into Microsoft Excel. The information obtained from the included studies was tabulated under the following: first name of the author, the year of publication, country of study, patient age, sample size, study design, type of thyroid disease, sample collection methods, NIH Quality Assessment Score for articles (NIHQAS), taste receptor polymorphism, type of taste perception, and SNP site.

### 2.4. Quality Assessment

An assessment tool suitable for observational, cohort, cross‐sectional, and case‐control studies consisting of 14 questions by NIH [14] was employed. Each question has five options (Yes/No/Cannot determine/Not applicable/Not reported). The range of scores: 0–4, was categorized as *poor*; 5–9, as *fair*; and those between 10 and 14 as *good*. Two authors independently assessed the articles, and any issues were resolved through discussions and consensus. Meta‐analyses could not be performed due to variations in the taste receptors and SNPs in the included articles.

## 3. Results

**FIGURE 1:**
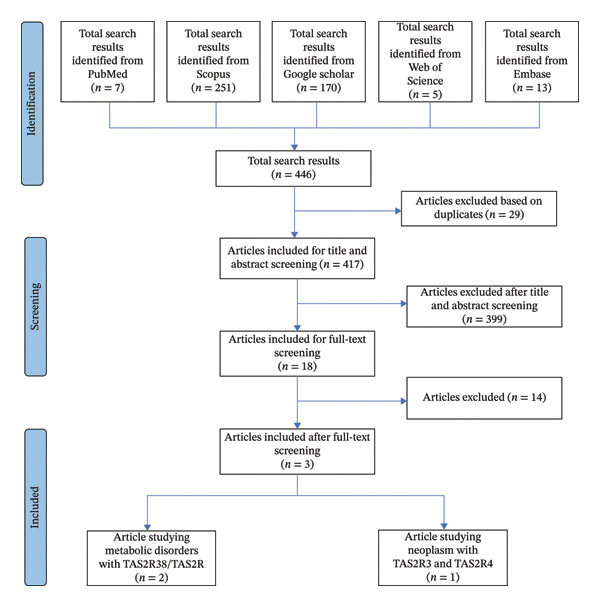
Identification of studies via databases.

A total of 446 articles were identified across five databases and were completed with manual search. This enabled us to find 7 articles from PubMed/Medline, 251 from Scopus, 5 from Web of Science, 170 from Google Scholar, and 13 from Embase. Duplicates were removed based on title and abstract, leaving 417 articles for review. After examining the FTs, 14 articles were excluded as irrelevant. The PRISMA‐ScR flowchart in Figure 1 illustrates the selection process. Most publications ranged from 2000 to 2025, with characteristics of the source of evidence. The current review included 3 articles that met the specified inclusion and exclusion criteria. A detailed summary of these articles is presented in Table 2.

**TABLE 2:** Characteristic features of the studies with quality assessment scores.

Authors/year	Study design	Country of research	Age range	Sample collection method	Lab investigation	Quality assessment criteria (NIH)
Costa et al. 2023 [15]	Cross‐sectional and observational study	Lisbon, Portugal	56.42 ± 13.83 years	Peripheral blood sample DXA scans	Standard assays for lipids, HbA1c, glucose, insulin, HOMA‐IR, uricemia, calcemia ELISA Spectrophotometry Endpoint SNP genotyping for TAS2R38 statistical	11

Clark et al. 2015 [7]	Experimental study, in vitro and in vivo	USA	56.42 ± 13.83 years; 46 ± 15 yrs for men and 45 ± 15 yrs for women	Human thyroid tissues obtained from surgeries Mouse thyroid gland HEK293 cells transfected with TAS2R	RT‐PCR, immunohistochemistry, functional assay, in situ hybridization, calcium imaging, transfection of HEK293 cells, thyroid hormone assay (T3/T4)	11

Choi et al.2018 [16]	Case control	South Korea	48.9 ± 8.48 yrs	Peripheral blood samples Prediagnosis serum sample	SNP genotyping Linkage disequilibrium Haplotype analysis Biomarker assay	11

### 3.1. The Various Types of Study Design With Details

Studies were performed across three different countries: (1) conducted in the United States by Clark​ et al. [7], categorized as an experimental study with both in vitro and in vivo methods having 374 participants; with an age range of 46 ± 15 years for men and 45 ± 15 years for women; (2) a cross‐sectional study from Portugal by Cost et al. [15], with 342 participants, with an age range of 56.42 ± 13.83 years; (3) a case‐control study from Korea by Choi et al.’s [16] analysis of 250 cases of papillary thyroid carcinoma (PTC) and 513 controls, aged above 30 years. As the study designs included in the review were cross‐sectional or case‐control, temporality or causality cannot be established, and the results are susceptible to recall bias.

There was heterogeneity across studies regarding laboratory methods that included sample collection, genetic analysis/molecular, and varied functional assays performed by these authors. Costa et al. [15] and Choi et al. [16] obtained peripheral blood samples, while Clark et al. [17] procured HEK293 cells transfected with specific TAS2R gene to confirm receptor activation and calcium signaling. Human and animal tissue samples were also collected for the experiments. SNP genotyping was performed by Costa et al. [15] and Choi et al. [16]. Additionally, linkage disequilibrium mapping and haplotype analysis targeting TAS2R3 and TAS2R4 genes to identify the association between genetic variations and thyroid cancer susceptibility were also studied [16]. Clark et al. [17] employed RT‐PCR to evaluate TAS2R gene expression in thyroid tissues and IHC to localize TAS2R proteins within the follicular cells. In situ hybridization was further used to confirm receptor expression at the cellular level. Various assays such as calcium imaging, thyroid hormones (T3 and T4), and iodine influx and reflux were employed to assess TSH‐dependent thyroid activity. Metabolic markers and biochemical parameters, using ELISA spectrometry, were employed by Costa et al. [15]. Both researchers [15, 16] measured the thyroid hormone levels (Tables 2 and 3).

**TABLE 3:** Overview of the functional role of various bitter taste receptors and agonists in regulating thyroid hormone synthesis.

Functional role in regulating thyroid hormone	Cells/tissue/disease	Agonists and varied TAS2R	Reference
TSH ligands inhibited both TSH‐dependent Ca2+ and iodine efflux.	Cell lines: Human, Nthy‐ori3‐1 human thyrocyte line	Camphor, denatonium benzoate, colchicine‐TAS2R4	[7]
		Camphor, chloramphenicol, cycloheximide, denatonium benzoate‐TAS2R10	
		6‐n‐propylthiouracil (PROP)‐ TAS2R 38	
		Chloramphenicol, denatonium benzoate‐TAS2R43	

TSH ligands inhibited both TSH dependent Ca2+ and iodine efflux/influx	Hypothyroidism	6‐n‐propylthiouracil (PROP)‐TAS2R 38	[15]
	Hyperthyroidism	Brassica family vegetables‐AVI haplo/diplo types	

Inhibits TSH‐Ca2+ signaling, iodide efflux	Papillary thyroid cancer	Camphor, colchicine‐TAS2R3 &4 (CC haplo type)	[16]

Regulates TSH levels, suppresses inflammatory mediators	Autoimmune diseases of the thyroid with stable ischemic heart disease	Resveratrol‐TAS2R14	[13]

### 3.2. Quality Assessment of Included Studies

Three articles were included, ranging from good to fair quality as per the criteria laid by the NIH quality assessment tool (Supporting file: S2, Table 2). Most of the studies did not provide detailed evidence of the various confounders and the clarity on the reporting on the loss of follow‐up rates.

### 3.3. Type of Taste Perception, Various Taste Receptors, Taste Receptors With Polymorphisms, and Associated SNP Site

**FIGURE 2:**
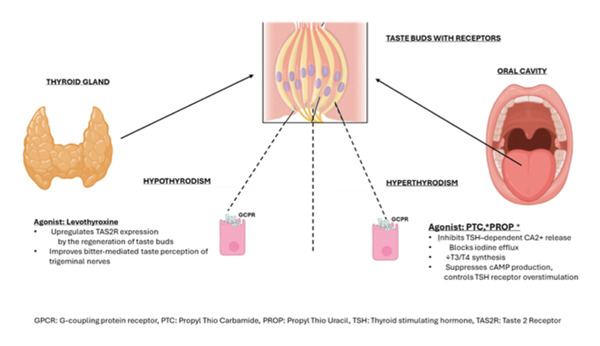
Influence of thyroid gland dysfunction on taste receptors.

Studies have shown that the bitter taste perception is strongly associated with thyroid dysfunction. Variants of TAS2Rs and their corresponding agonist in modulating thyroid hormones have been demonstrated by Clark et al. [17], Costa et al. [15], and Choi et al. [16]. Polymorphism in TAS2R 38 categorizes patients into supertasters, nontasters, and intermediates [15]. TAS2R 3&4 receptor polymorphism is associated with a lower risk of PTC [16] (Tables 3 and 4, Figure 2).

**TABLE 4:** Overview of the association of thyroid diseases and various bitter taste receptors, gene polymorphism, and their clinical functions.

Taste receptor	Gene polymorphisms (SNPs)	Functional/taste implication	Thyroid dysfunction associations	Reference
TAS2R38	P49A, rs713598 (alanine to proline substitution)	Super‐tasters: PAV/PAV	PAV genotype is protective against thyroid dysfunction	[15]
	A262I, rs1726866 (valine to alanine substitution)	Non‐tasters: AVI/AVI	Val/Val at A262V increases hypothyroidism & hyperthyroidism risk	
	V296I, rs10246939 (isoleucine to valine substitution)	Intermediate: PAV/AVI		
	Haplotypes: PAV, AVI, PVV, AVV			

TAS2R42	S196F, rs5020531 (serine to phenylalanine substitution)	Taste ligand unknown	Strongly associated with elevated free T4 and T3 levels	[7]

TAS2R38	—	—	Nthy‐Ori 3–1 cells with nontaster TAS2R38 variant unresponsive to PROP in calcium signaling	[7]

TAS2R3	C⟶T, (Synonymous) rs2270009 and rs2234001 (Cytosine to thymine),	Coding variation is likely to alter receptor function and ligand sensing	The CC haplotype is 40% lower risk of papillary thyroid carcinoma	[16]

TAS2R4	Val96Leu, rs2270009 and rs2234001 (Valine to leucine),	Coding variation is likely structural/stability changes in receptor function	CC haplotype is associated with ↓ total T3 (TT3) levels, which may mediate the reduced PTC risk	[16]

## 4. Discussion

### 4.1. The Taste Receptors

The taste receptors in oral and extraoral locations are stimulated by the different taste ligands including sweet, salty, bitter, and umami [18, 19]. Among these, TAS2Rs are linked to bitter ligands. TAS2R plays a crucial role in warding off dangerous compounds that may be inhaled or consumed [5, 6]. TAS2Rs have been suggested to influence thyroid‐related pathways, primarily through experimental models; however, the exact mechanisms and clinical relevance remain under investigation. The human genome encodes 25 different isoforms of TAS2Rs, a subgroup of G protein coupling receptors (GPCRs). In addition to being found on the tongue’s taste buds, they are also found in extraoral locations such as the respiratory and gastrointestinal tracts [20–26]. Polymorphism in these receptors has been seen in recent years, and it may be linked to infections, autoimmune diseases, or malignancies. These taste receptor proteins are encoded by genes found on Chromosomes 5p, 7q, and 12p [27].

### 4.2. TAS2R and TSH

Maturation, proliferation, and thyroglobulin endocytosis from the colloid are among the functions of TSH, which is essential for preserving the homeostasis of the thyroid and other tissues. The efflux of iodine, which is oxidized by thyroid peroxidase (TPO) prior to being incorporated into thyroglobulin, a crucial component in the synthesis of triiodothyronine (T3) and thyroxine (T4), is mediated by Ca2+‐dependent TSH receptors. TPO antagonists and agonists both affect these secretory thyroid hormones [28, 29]. In addition to these, the functional role of how taste stimuli are perceived by various agonists in modulating thyroid hormones, also the various pathways by which the extraoral taste receptors are activated, contributes to this process and needs further research [30, 31]. Antithyroid drugs such as methimazole, PTC, and PROP are a few examples of TPO inhibitors that activate TAS2R receptors and clinically reduce the excess thyroid hormone production [5, 6, 15, 31–33].

Activation of the TAS2R38 gene agonists has made it possible to classify people into distinct taste receptor phenotypes according to different genotype combinations. The heterozygous PAV/AVI diplotype is linked to intermediate sensitivity, the PAV/PAV type has the highest sensitivity, and the AVI/AVI diplotype, which is known as a nontaster, has the lowest sensitivity. Research has also demonstrated that the TAS2R38 receptors influence naturally occurring substances including goitrin and sinigrin [34, 35].

Patients with hypothyroidism who receive levothyroxine are reported to have an improvement in perceiving bitter tastes better [36]. According to a comparative study, Asian populations perceive PTC/PROP nontasters at a rate between 4.6% and 9.7% lower than that of European populations [37].

A retrospective investigation revealed that activation of certain TAS2R receptors, including TAS2R4, TAS2R10, and TAS2R42, affects the signaling pathway of TSH and modifies it. This alteration inhibits iodine influx and decreases intercellular Ca2+ release, both of which are essential for the production of T3 and T4. Humans with the TASR42 polymorphism (SNP rs5020531) exhibit reduced thyroid hormone levels. Reduced iodine incorporation into precursor thyroglobulin was associated with a notable rise in free T3 (fT3) and free T4 (fT4) levels. It is necessary to investigate the functional role of TASR42 because it is regarded as an orphan receptor [16, 17].

### 4.3. TAS2R and Anti‐Inflammatory Response

The TAS2R38 detects acyl‐homoserine lactones (AHLs) to stimulate calcium‐dependent nitric oxide (NO) secretion and its functional role as a sentinel receptor to identify bacteria and regulate innate immune responses has been highlighted [38–40]. TAS2R receptors most importantly hTaS2R 38, are known to have immunosuppressive activities by inhibiting IgE‐dependent mast cell degranulation. Exposure of immune surveillance cells, macrophages, and monocytes to bitter compounds from the extracts of goitrin or bupleuri radix is said to decrease the synthesis of cytokines, viz., TNF‐α, which influence inflammation. This activity was not functional in TAS2R diplotype, AVI/AVI type [41, 42]. These observations suggest a possible immunomodulatory role of TAS2Rs; however, their relevance to thyroid‐related inflammation in humans remains to be established.

### 4.4. TAS2R and Autoimmune Disease

According to an analysis of the gene expression omnibus on TAS2R genes, people who may or may not have Hashimoto’s thyroiditis clinically had differential expression of TAS2R16 and TAS2R42 [43]. People with PAV haplotypes are linked to a higher immunological response. It is claimed that TAS2R activation lessens cytokine storms in thyroid‐related autoimmune diseases [17, 44].

TAS2R38 is expressed in neutrophils, macrophages, connective tissue, and component innate immune cells [39, 45]. Lymphocytes, which are among the most crucial elements of adaptive immunity, express TAS2R38 similarly to neutrophils and macrophages. Increased expression of this receptor has been demonstrated in several lymphocyte subpopulations, including CD8+ cytotoxic cells, native, central, and effector cells [41]. Pharmacological agents such as resveratrol decrease proinflammatory cytokines such as TNF‐alpha, IL‐6, and nuclear factor Kappa B (NK‐κB) by activating receptors on TAS2R14 [12, 44, 45].

### 4.5. Association Between TAS2R and Thyroid Cancer

The TAS2R 3/4 CC haplotype was linked to lower T3 levels and protected against PTC in a Korean study. The authors hypothesized that altered chemosensation of exogenous toxins and reduced stimulatory activity of T3 may provide anticarcinogenic protection, albeit the exact mechanisms must be clarified [16].

### 4.6. Benefits of Pharmacological Agents in Thyroid Dysfunction

Preliminary hypothesis suggests that TAS2Rs could represent potential future therapeutic targets for regulating thyroid activity and treating comorbidities associated with thyroid dysfunctions, as current evidence remains sparse. Identifying extensive haplotype diversity of TAS2Rs in human populations may offer the potential for personalized therapies, guiding individuals in consuming as well as the ability of many pharmaceuticals to activate TAS2Rs [46].

## 5. Limitations

The majority of studies focus on the bitter taste, with a particular emphasis on TAS2R receptors. Although the thyroid gland can influence multiple taste receptors, such as TAS1R (sweet, umami) and ENAC (salt), there is a lack of studies on these receptors. The mechanism by which thyroid dysfunction alters salivary flow and composition, as well as its influence on TAS2R expression in salivary glands, needs to be explored. We observed heterogeneity in the quality of publications. Hence, a large number of high‐quality multicentric studies with large sample sizes and longer follow‐up periods, taking into consideration various comorbidities as well as cultural aspects across countries, pave the way for validating TAS2R receptors and their agonists as well as for screening. Future studies may explore whether TAS2R38 PAV/AVI phenotype individuals are at risk of developing thyroid dysfunction.

## 6. Conclusion and Future Perspectives

This scoping review evaluated the existing literature exploring the relationship between bitter taste receptor (TAS2R) polymorphisms with thyroid dysfunction and associated taste disturbances. The available evidence is extremely limited and largely derived from experimental studies and a small number of observational studies. Consequently, the current review does not permit causal inferences or clinical generalizations regarding the role of TAS2Rs in thyroid‐related dysgeusia. This review aims to integrate the current understanding of the genetic variants and pathophysiological links between thyroid dysfunction and dysgeusia. Recognizing taste disturbances as a relevant marker of endocrine imbalance encourages more holistic patient care. Due to paucity of available research, the proposed link between TAS2Rs and thyroid disorders could be novel therapeutic targets for treating hypo‐ or hyperthyroidism, as it remains hypothetical, highlighting the research gap, and it points toward an intriguing future direction, addressing associated sensory changes which can improve clinical outcomes and QOL. TAS2Rs may act as guarding receptors for ingested or endogenous bitter compounds, modulating thyroid function as a protective mechanism against toxins. Identification of gene polymorphisms helps in educating patients with genetic variants (e.g., TAS2R38‐AVI) with increased hyperthyroidism susceptibility, coupled with intervention programs that in turn help in improving the QOL for patients with thyroid diseases.

## Author Contributions

Conceptualization: S.C., S.N., and C.C.; methodology: S.C., P.P., and A.C.; formal analysis: S.C. and S.N.; investigation: G.R. and C.C.; data curation: P.P., C.C., and A.C.; writing–original draft preparation: S.C., S.N., and C.C.; writing–review and editing: G.R., S.C., and S.N.; visualization: C.C. and A.C.; supervision: S.C. and G.R.; project administration: G.R.; funding acquisition: no funding was granted for the conduction of this work.

## Funding

This work did not receive any specific grant from funding agencies.

## Ethics Statement

The systematic review is based on the published articles, hence ethical committee approval is not necessary.

## Conflicts of Interest

The authors declare no conflicts of interest.

## Supporting Information

Additional supporting information can be found online in the Supporting Information section.

## Supporting information


**Supporting Information 1** Supporting File 1: Search strategy.

**Supporting Information 2** Supporting File 2: Risk of bias, NIH checklist.

## Data Availability

The data that support the findings of this study are available on request from the corresponding author. The data are not publicly available due to privacy or ethical restrictions.
