# Supplementary material for: Endocrine‐Taste Crosstalk: A Scoping Review on Thyroid Dysfunction and Its Genetic Links to Taste Receptors With Dysgeusia
Source: Int J Endocrinol. 2026 Apr 1;2026:1156681. doi: 10.1155/ije/1156681 (PMC13045250; doi:10.1155/ije/1156681)
Supplement: Supplementary file 1 — Supporting Information 1 Supporting File 1: Search strategy. [file IJE-2026-1156681-s001.docx]

SUPPLEMENTARY FILE 1: SEARCH STRATEGY

| ID | PubMed | Results |
| --- | --- | --- |
| #1 | "dysgeusia" OR "dysgeusias" OR "altered taste" OR "taste dysfunction" OR "taste disorder" OR "taste perception" OR "ageusia" OR "hypogeusia" OR "parageusia" |  |
| #2 | "thyroid dysfunction" OR "thyroid dysfunctionality" OR "thyroid disease" OR "thyroid disorders" OR "hypothyroidism" OR "hyperthyroidism" OR "autoimmune thyroiditis" OR "Hashimoto's thyroiditis" OR "Graves' disease" OR "thyroid cancer" OR "thyroid carcinoma" OR "thyroid neoplasm" OR "thyroid abnormalities" |  |
| #3 | "taste receptor" OR "taste receptors" OR "taste receptor gene" OR "taste receptor genes" OR TAS1R OR TAS2R OR "bitter taste" OR "sweet taste" OR “umami taste” OR "taste gene" OR "taste genes" OR "taste signaling" OR "taste pathway" OR "gene polymorphism" OR "gene polymorphisms" OR "genetic variation" OR "genetic variations" OR SNP OR SNPs |  |
| #4 | #1 AND #2 AND #3 | 7 |
|  | Scopus |  |
| #1 | "dysgeusia" OR "dysgeusias" OR "altered taste" OR "taste dysfunction" OR "taste disorder" OR "taste perception" OR "ageusia" OR "hypogeusia" OR "parageusia" |  |
| #2 | "thyroid dysfunction" OR "thyroid dysfunctionality" OR "thyroid disease" OR "thyroid disorders" OR "hypothyroidism" OR "hyperthyroidism" OR "autoimmune thyroiditis" OR "Hashimoto's thyroiditis" OR "Graves' disease" OR "thyroid cancer" OR "thyroid carcinoma" OR "thyroid neoplasm" OR "thyroid abnormalities" |  |
| #3 | "taste receptor" OR "taste receptors" OR "taste receptor gene" OR "taste receptor genes" OR TAS1R OR TAS2R OR "bitter taste" OR "sweet taste" OR “umami taste” OR "taste gene" OR "taste genes" OR "taste signaling" OR "taste pathway" OR "gene polymorphism" OR "gene polymorphisms" OR "genetic variation" OR "genetic variations" OR SNP OR SNPs |  |
| #4 | #1 AND #2 AND #3 | 251 |
|  | Google scholar |  |
| #1 | "dysgeusia" OR "taste dysfunction" OR "taste disorder" OR "altered taste" |  |
| #2 | "thyroid dysfunction" OR "thyroid disease" OR "hypothyroidism" OR "hyperthyroidism" OR "autoimmune thyroiditis" OR "Hashimoto's thyroiditis" OR "Graves' disease" |  |
| #3 | "taste receptor gene" OR "taste receptor" OR TAS1R OR TAS2R OR "sweet taste" OR "bitter taste" OR "gene polymorphism" OR "genetic variation" |  |
| #4 | #1 AND #2 AND #3 | 170 |
|  | Web Of Science |  |
| #1 | "dysgeusia" OR "dysgeusias" OR "altered taste" OR "taste dysfunction" OR "taste disorder" OR "taste perception" OR "ageusia" OR "hypogeusia" OR "parageusia" |  |
| #2 | "thyroid dysfunction" OR "thyroid dysfunctionality" OR "thyroid disease" OR "thyroid disorders" OR "hypothyroidism" OR "hyperthyroidism" OR "autoimmune thyroiditis" OR "Hashimoto's thyroiditis" OR "Graves' disease" OR "thyroid cancer" OR "thyroid carcinoma" OR "thyroid neoplasm" OR "thyroid abnormalities" |  |
| #3 | "taste receptor" OR "taste receptors" OR "taste receptor gene" OR "taste receptor genes" OR TAS1R OR TAS2R OR "bitter taste" OR "sweet taste" OR “umami taste” OR "taste gene" OR "taste genes" OR "taste signaling" OR "taste pathway" OR "gene polymorphism" OR "gene polymorphisms" OR "genetic variation" OR "genetic variations" OR SNP OR SNPs |  |
| #4 | #1 AND #2 AND #3 | 5 |
|  | Embase |  |
| #1 | "dysgeusia" OR "dysgeusias" OR "altered taste" OR "taste dysfunction" OR "taste disorder" OR "taste perception" OR "ageusia" OR "hypogeusia" OR "parageusia" |  |
| #2 | "thyroid dysfunction" OR "thyroid dysfunctionality" OR "thyroid disease" OR "thyroid disorders" OR "hypothyroidism" OR "hyperthyroidism" OR "autoimmune thyroiditis" OR "Hashimoto thyroiditis" OR "Graves disease" OR "thyroid cancer" OR "thyroid carcinoma" OR "thyroid neoplasm" OR "thyroid abnormalities" |  |
| #3 | "taste receptor" OR "taste receptors" OR "taste receptor gene" OR "taste receptor genes" OR TAS1R OR TAS2R OR "bitter taste" OR "sweet taste" OR "umami taste" OR "taste gene" OR "taste genes" OR "taste signaling" OR "taste pathway" OR "gene polymorphism" OR "gene polymorphisms" OR "genetic variation" OR "genetic variations" OR SNP OR SNPs |  |
| #4 | #1 AND #2 AND #3 | 13 |
