# Supplementary material for: Endocrine‐Taste Crosstalk: A Scoping Review on Thyroid Dysfunction and Its Genetic Links to Taste Receptors With Dysgeusia
Source: Int J Endocrinol. 2026 Apr 1;2026:1156681. doi: 10.1155/ije/1156681 (PMC13045250; doi:10.1155/ije/1156681)
Supplement: Supplementary file 2 — Supporting Information 2 Supporting File 2: Risk of bias, NIH checklist. [file IJE-2026-1156681-s002.docx]

| **NIH Quality Assessment for experimental studies ,[7]** | | | | | | | | | | | | | | | | | |
| --- | --- | --- | --- | --- | --- | --- | --- | --- | --- | --- | --- | --- | --- | --- | --- | --- | --- |
| Author & Year | Q1 | Q2 | Q3 | Q4 | Q5 | Q6 | Q7 | Q8 | | Q9 | Q  10 | Q  11 | Q  12 | Q  13 | Q  14 | Total | Summary quality |
| A7. Clark &2015 | Y | Y | Y | Y | Y | NA | Y | Y | NR | | NA | Y | Y | Y | Y | 11 | Good quality |

**Supplementary file2: NIH Quality assessment of the articles**

Legend= Yes, N=No, NR=Not reported, NA= Not Applicable, CD= Cannot determine, [7]: article no.7

| **NIH Quality Assessment Tool for Observational Cohort and Cross-Sectional Studies**,[15] | | | | | | | | | | | | | | | | | |
| --- | --- | --- | --- | --- | --- | --- | --- | --- | --- | --- | --- | --- | --- | --- | --- | --- | --- |
| Author & Year | Q1 | Q2 | Q3 | Q4 | Q5 | Q6 | Q7 | Q8 | Q9 | Q  10 | Q  11 | Q  12 | Q  13 | Q  14 | Total | Summary quality |  |
| A26. Costa&  2023 | Y | Y | Y | Y | N | Y | Y | Y | Y | NR | NA | Y | Y | Y | 11 | Good quality |  |

Legend= Yes, N=No, NR=Not reported, NA= Not Applicable, CD= Cannot determine, [15] :article no15

| **NIH Quality Assessment Checklist for Case-Control Studies[16]** | | | | | | | | | | | | | | | | |
| --- | --- | --- | --- | --- | --- | --- | --- | --- | --- | --- | --- | --- | --- | --- | --- | --- |
| Author& Year | Q1 | Q2 | Q3 | Q4 | Q5 | Q6 | Q7 | Q8 | Q9 | Q  10 | Q  11 | Q  12 | Q  13 | Q  14 | Total | Summary quality |
| A34.Choi, J. H&2018 | Y | Y | Y | Y | N | Y | Y | Y | NR | Y | NA | Y | Y | Y | 11 | Good quality |

Legend= Yes, N=No, NR=Not reported, NA= Not Applicable, CD= Cannot determine, [16]: article no16

| **NIH Quality Assessment Checklist for Pre-Post Intervention Studies[17]** | | | | | | | | | | | | | | | | | |
| --- | --- | --- | --- | --- | --- | --- | --- | --- | --- | --- | --- | --- | --- | --- | --- | --- | --- |
| Author & Year | Q1 | Q2 | Q3 | Q4 | Q5 | Q6 | Q7 | Q8 | Q9 | Q  10 | Q  11 | Q  12 | Q  13 | Q  14 | Total | Summary quality |  |
| Chekalina, N.&2017 | Y | Y | CD | R | Y | Y | CD | Y | N |  | NR | N | NR | Y | 6 | Fair quality |  |

Legend= Yes, N=No, NR=Not reported, NA= Not Applicable, CD= Cannot determine, [17] : article no 17
